# Long-term channelrhodopsin-2 (ChR2) expression can induce abnormal axonal morphology and targeting in cerebral cortex

**DOI:** 10.3389/fncir.2013.00008

**Published:** 2013-01-31

**Authors:** Toshio Miyashita, Yu R. Shao, Jason Chung, Olivia Pourzia, Daniel E. Feldman

**Affiliations:** Department of Molecular and Cellular Biology, Helen Wills Neuroscience Institute, University of California BerkeleyBerkeley, CA, USA

**Keywords:** optogenetics, *in utero* electroporation, axon development, circuit

## Abstract

Long-term expression of optogenetic proteins including channelrhodopsin-2 (ChR2) is widely used to study neural circuit function, but whether ChR2 expression itself perturbs circuits is not known. We expressed a common construct, CAG::ChR2 (H134R)-EYFP-WPRE, in L2/3 pyramidal cells in rat somatosensory cortex via *in utero* DNA electroporation (IUE). L2/3 pyramidal cells expressed ChR2-EYFP, but histology revealed abnormal morphology and targeting of ChR2-EYFP expressing axons, beginning at postnatal day (P) 33 and increasing with age. Axonal abnormalities included cylinders that enveloped pyramidal cell proximal apical dendrites, and spherical, calyx-like structures that surrounded neuronal cell bodies, including in L4. These are abnormal subcellular and laminar targets for L2/3 pyramidal cell synapses. Abnormalities did not occur in cells expressing GFP instead of ChR2, or in intermixed ChR2-negative axons. Long-term viral-mediated expression (80 d) did not cause axonal abnormalities when the CAG promoter was used, but produced some abnormalities with the stronger αCaMKII promoter (albeit much less than with *in utero* electroporation). Thus, under some circumstances high-level, long-term expression of ChR2-EYFP can perturb the structural organization of cortical circuits.

## Introduction

Optogenetics tools including channelrhodopsin-2 (ChR2) provide a powerful approach to assay and manipulate neural circuit function, and promise new therapeutic interventions for neurological disease (Zhang et al., 2007; Bernstein and Boyden, 2011; Fenno et al., 2011). A critical prerequisite of optogenetics is that expression of optogenetic proteins does not, in itself, alter neural circuit function. This is a particular concern for long-term and/or high-level expression under strong promoters, which is common to achieve efficient light activation of neurons (e.g., Huber et al., 2008; Toni et al., 2008; Petreanu et al., 2009; Adesnik and Scanziani, 2010). The effects of high-level, long-term expression of ChR2 on circuit wiring or function are unclear.

While developing methods to use ChR2 to study the function of layer (L) 2/3 pyramidal cells in the whisker region of somatosensory cortex, we noticed an apparent abnormal effect of long-term, high-level ChR2 expression on cortical microcircuits. We expressed ChR2 (H134R) fused to EYFP (ChR2-EYFP) in L2/3 pyramidal cells in rat somatosensory cortex (S1) via *in utero* electroporation (Saito and Nakatsuji, 2001; Tabata and Nakajima, 2001; Miyashita et al., 2010) under the CAG promoter, as used in several prior studies of S1 circuit function (Huber et al., 2008; Adesnik and Scanziani, 2010). This drove strong ChR2-EYFP expression in L2/3 pyramidal cells, and robust spiking induced by blue light *in vivo*. However, long-term expression (≥40 days) caused major abnormalities in axonal morphology in ChR2-EYFP expressing neurons, including formation of structures suggestive of abnormally large, incorrectly targeted synapses. These abnormalities did not require ChR2 activation (i.e., they occurred in animals raised in normal room lighting), and did not occur in intermixed ChR2-negative axons or in cells expressing GFP instead of ChR2. Substantially fewer axonal abnormalities were observed with long-term viral-mediated expression (80 days), and their level depended on the promoter (they were present with the strong αCaMKII promoter, but not the weaker CAG promoter). Thus, high-level, long-term expression of ChR2 has the potential to alter axonal morphology and targeting in cerebral cortex.

## Methods

All animal experimental procedures were approved by the UC Berkeley Animal Care and Use Committee, and conform to standards of the National Institutes of Health.

### *In utero* electroporation of ChR2

The pCAG-ChR2 (H134R)-EYFP-WPRE plasmid for *in utero* electroporation was constructed as follows. First, *Bam*HI/*Eco*RI ChR2-H134R-EYFP-WPRE DNA fragment from FCK1.3-hChR2-EYFP-WPRE (kindly provided by K. Deisseroth, Stanford University) was subcloned into pCDNA3. Second, polymerase chain reaction (PCR) amplification was performed using pCDNA3-hChR2-EYFP-WPRE plasmid DNA as a template with the following set of oligonucleotide primers: 5′-TAA ACC GGT GCC ACC ATG GAC TAT G-3′, and 5′-TCG CGT CGA CCA TTT AGG TGA CAC TAT AG-3′. Third, the amplified DNA fragment was subcloned into *Age*I/ *Xho*I of pCAGGS vector (Niwa et al., 1991).

Timed-pregnant Long-Evans rats (18 days post coitum) were anesthetized with isoflurane (5% induction, 2% maintenance). The uterus was lifted from the abdominal cavity, and embryos were visualized and electroporated through the uterine wall. A glass pipette (30–40 μm tip diameter) was introduced into the left lateral cerebral ventricle, and ~1 μL of plasmid DNA solution was pressure injected. The DNA solution contained: pCAG-ChR2 (H134R)-EYFP-WPRE plasmid (1 μg/μL), pCAG-DsRed plasmid (0.5 μg/μL), pCAG-GFP (0.5 μg/μL) plasmid, and 0.05% Fast Green. The capillary was removed, and forceps with round electrodes (CUY650-5; NEPA GENE) were placed on either side of the head, outside the uterine wall. Five 50 V square pulses (50 ms duration, 950 ms interval) were delivered via a square pulse electroporator (BTX ECM830, Harvard Apparatus). The uterus was returned to the abdominal cavity, and the abdominal wall and skin were sutured. Buprenorphine was given for post-operative analgesia (0.05 mg/kg, subcutaneously, twice at 8 h interval). Pups were born by natural delivery.

The purpose of the GFP plasmid was to increase overall fluorescence intensity to enable identification of expressing newborn pups by transcranial imaging at P0. Only pups showing strong green (GFP and EYFP) fluorescence in S1 on P0 were used in later experiments. The purpose of the DsRed plasmid was to enable fluorescently-targeted patching of expressing cells during brain slice physiology (GFP fluorescence was not imaged during physiology experiments, to avoid activating ChR2 with GFP excitation wavelengths). 61% of green fluorescent cells also expressed DsRed, and all DsRed cells expressed ChR2 (see “Results”), indicating that individual cells take up multiple plasmids.

### Viral expression of ChR2

For virus-mediated expression of ChR2, rats 30–40 days of age were anesthetized by either ketamine/xylazine (90 and 10 mg/kg, i.p.) or isoflurane (5% induction, 2% maintenance, in 2 L/min O_2_), and placed in a stereotaxic frame. The scalp was incised and a 3–4 mm craniotomy made over S1 (2 mm rostral, 5.5 mm lateral from bregma). Virus was either adeno-associated virus 2 serotype 5 (AAV2.5) expressing ChR2-EYFP under the CAG promoter (1 μL, rAAV5/CAG-ChR2-GFP, 3 × 10^12^/mL; Gene Therapy Center virus vector core, Univ. of North Carolina), AAV2.5 expressing ChR2-EYFP under the αCamKII promoter (1 μL, AAV2.5-αCamKII:ChR2 (H134R)-EYFP-WRPE, 3 × 10^12^/mL; gift of Dr. Karl Deisseroth, Stanford University), or replication-incompetent lentivirus expressing ChR2-EYFP under the αCamKII promoter (0.2–0.3 μL, Lenti-αCamKII:ChR2 (H134R)-EYFP-WRPE, 2.65 × 10^9^/mL). Virus was pressure injected into S1 at a depth of 400–800 μm. The craniotomy was sealed with silicon elastomer, and the scalp closed with sutures and Vetbond. Rats received buprenorphine for post-operative analgesia (0.05 mg/kg subcutaneously, twice at 8 h interval).

### BDA labeling of callosal S1 axons

To label callosal S1 axons projecting to S1, age rats that were expressing ChR2-EYFP from prior *in utero* electroporation in left S1 were anesthetized with isoflurane. Biotinylated dextran amine (BDA) (10,000 MW, 10 mg/mL; Invitrogen) was injected via a craniotomy into right S1 to anterogradely label callosal axons in the left S1. The craniotomy was sealed with silicon elastomer, and the skin closed with sutures and Vetbond. Buprenorphine was given for post-operative analgesia (0.05 mg/kg subcutaneously, twice at 8 h interval).

### Recording light-evoked neural responses *In vivo* and *In vitro*

For *in vivo* recordings, rats were anesthetized with urethane (1.5 g/kg i.p.), and a craniotomy was made over left S1 (3–4 mm diameter, centered 2 mm caudal and 5.5 mm lateral from Bregma). A small slit was made in the dura. Multiunit extracellular recordings were made with tungsten electrodes (5 MΩ at 1 kHz) at 3000× gain, filtered at 0.5–3 kHz band pass, and digitized at 32 kHz with 12-bit resolution. Data was collected using custom routines in Igor Pro (WaveMetrics). Anesthesia was maintained with supplemental urethane (10% of original dose, i.p.) and body temperature was maintained at 37°C. ChR2 was activated with a 473 nm laser (Shanghai Laser and Optics Company, BL473T3) coupled to a multi-mode optical fiber (0.2 mm diameter). The fiber was positioned ~5 mm above the craniotomy. Light was delivered in pulses (1–2 ms, presented in 20–50 Hz trains, 400 ms train duration).

For *in vitro* recordings, acute brain slices (400 μm thick) were prepared by standard methods (Allen et al., 2003). Whole-cell recordings were targeted to ChR2-expressing neurons in S1, identified by DsRed or EYFP fluorescence (Rolera XR camera, Q imaging software). Recordings were made at 31°C using K gluconate internal (mM: 116 K gluconate, 20 HEPES, 6 KCl, 2 NaCl, 0.5 EGTA, 4 MgATP, 0.3 NaGTP, 5 Na_2_phosphocreatine, pH 7.2, 295 mOsm). External solution contained (mM): 119 NaCl, 26.2 NaHCO_3_, 11 D−(+)-glucose, 1.3 MgSO_4_, 2.5 KCl, 1 NaH_2_PO_4_, 2.5 CaCl_2_. A 443-nm blue laser (40 mW, CrystaLaser DL445-040) was coupled via a multimodal fiber to the microscope epifluorescence arm, and projected to the slice through a 4× air objective with beam diameter 312 μm. Laser intensity and timing were controlled by analog voltage commands generated in IGOR Pro.

### Perfusion and histology

Animals were deeply anesthetized with isoflurane, and perfused transcardially with 0.9% NaCl followed by 4% paraformaldehyde (PFA) in 0.1 M phosphate buffer (PB, pH 7.4, 15 min). Brains were removed, post-fixed for 2 h in 4% PFA in 0.1 M PB, and sunk in 30% sucrose in 0.1 M PB for cryoprotection (20–40 h at 4°C). Brains were sectioned (50 μm) in the coronal plane on a freezing microtome.

For viral-mediated expression cases, the EYFP/GFP signal was amplified by immunostaining with anti-GFP antibody. This was not necessary in IUE cases, where native expression was substantially stronger. Free-floating sections were incubated in blocking solution (0.5% Triton X-100 and 5% normal goat serum in phosphate-buffered saline, PBS) for 1 h at room temperature, followed by 40–48 h incubation at 4°C with rabbit anti-GFP antibody (1:2000, MBL) in blocking solution. Sections were rinsed with PBS, and incubated for 90 min at room temperature in secondary antibody solution (Alexa-488 conjugated goat anti-rabbit, 1:200 in blocking solution, Invitrogen). Sections were washed in PB 3 times for 5 min each, and mounted in Vectashield. For double immunostaining, additional primary antibody was included (mouse anti-NeuN, 1:400, Millipore, MAB377; rabbit anti-Collagen type IV, 1:200, Abcam, AB6586; or mouse antibody SMI32, 1:200, Abcam, AB73273), and appropriate secondary was used (Alexa-488 or 594 conjugated goat anti-mouse or -rabbit).

To visualize BDA-labeled axons in S1, free-floating sections were incubated for 1 h in blocking solution, followed by 90 min at room temperature in Alexa 647-conjugated streptavidin (Invitrogen; 1:200 dilution).

### Confocal microscopy

Fluorescently labeled tissue sections were scanned by confocal microscopy (Zeiss LSM 710 Axio Observer, Carl Zeiss). Images in figures are maximum Z-projections from sequential optical sections (200–300 nm intervals). Image orientation, size, brightness, and contrast were adjusted uniformly across the entire image frame, using ZEN 2009 light edition software (Carl Zeiss). For analysis of relative ChR2 expression level, confocal scans were made in L2/3 at the region of maximal expression using a Zeiss LSM 780 confocal with 32-channel GaAsP spectral detector, and EYFP signal intensity was separated from GFP or Alexa-488 by linear spectral unmixing using Zen software (Zeiss).

## Results

### Expression of ChR2 by IUE

**Figure 1:**
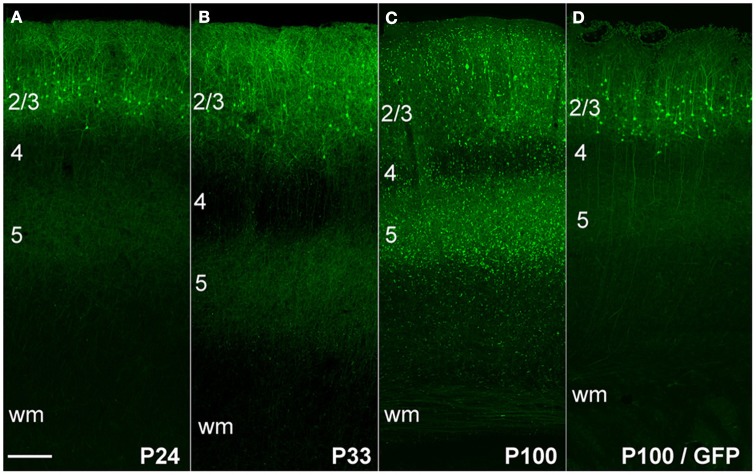
**ChR2 expression in L2/3 pyramidal cells, as a function of age after *in utero* electroporation. (A)** ChR2 expression in a P24 rat showing normal somatodendritic and axonal morphology of L2/3 pyramidal cells, including axon branches in L5. **(B** and **C)** Increasing appearance of abnormal EYFP+ puncta at P33 and P100. **(D)** Absence of puncta in a rat electroporated with GFP instead of ChR2 (age: P100). Scale bar, 200 μm. Numbers indicate cortical layers. wm, white matter.

We first expressed ChR2 (H134R)-EYFP in L2/3 pyramidal cells by *in utero* DNA electroporation (IUE) at 18 d of gestation (E18). In these experiments, we co-electroporated three plasmids: pCAG-ChR2 (H134R)-EYFP, pCAG-GFP, and pCAG-DsRed, all using the CAG promoter. Electroporation was targeted to the left primary somatosensory cortex (S1). In separate animals, only pCAG-GFP was electroporated. IUE of either the ChR2-EYFP/GFP/DsRed combination, or of GFP alone, resulted in strong fluorescence in L2/3 pyramidal neurons in left S1 (Figure 1). Fluorescence was observed in the somatodendritic compartment of these neurons, as well as in their axons that innervate L2/3 and L5, leading to a characteristic double band of fluorescence in L2/3 and L5 of S1. This expression pattern matches prior studies of ChR2 or GFP expression in L2/3 pyramidal cells (Hatanaka et al., 2004; Huber et al., 2008; Adesnik and Scanziani, 2010).

**Figure 2:**
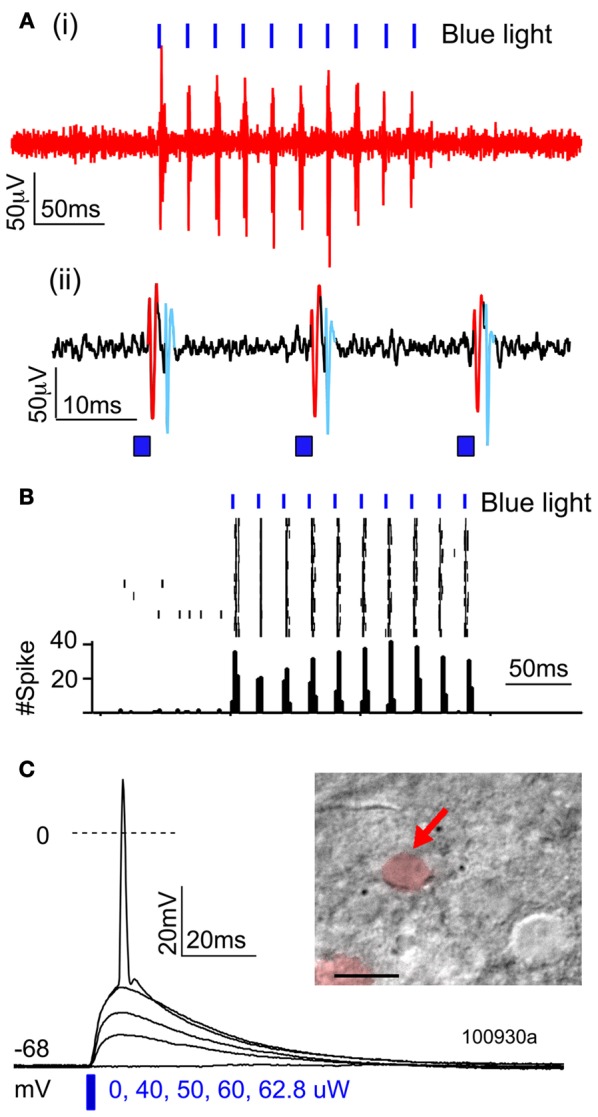
**Functional activation of ChR2 *in vivo* and *in vitro*. (A,B)** Extracellular multiunit recording in L2/3 *in vivo* (200–300 um depth) in a urethane-anesthetized rat (P100). **(A)** Raw voltage recording at two time scales. **(B)** Spikes elicited by trains of 2-ms light pulses (473 nm, 20 mW, 50 Hz, 10 s intertrial interval). **(C)** Light-evoked potentials recorded from a ChR2-expressing L2/3 neuron in a brain slice, in response to increasing light intensity (2-ms light pulse).

To confirm that ChR2 formed functional membrane channels, we first recorded light-evoked extracellular spikes in layer 2/3 of P100 rats (*n* = 3) that had undergone IUE of ChR2/GFP/DsRed. Rats were anesthetized, and multiunit recordings were made in layer 2/3 (200–300 μm below pia). Blue light from a 473 nm laser was flashed at the cortical surface via an optic fiber. Light pulses (1–2 ms duration, presented in trains at 20–50 Hz) evoked reliable spikes, and light-evoked firing showed little adaptation up to 50 Hz (Figures 2A,B), as expected for ChR2(H134R).

To quantify what fraction of green fluorescent neurons expressed ChR2, we used a combination of histology and whole-cell recordings in acute S1 slices. This was necessary because green fluorescence originated both from the ChR2-EYFP fusion protein and from cytosolic GFP, which was expressed from an independent plasmid. We first used two-color confocal microscopy in 50-μm histological sections to show that 61% of green fluorescent neurons co-expressed DsRed (1421/2135 neurons, 37 whisker-related columns, 10 rats). Next, we targeted whole-cell current clamp recordings to DsRed+ neurons in S1 slices (P17–21), and found that 100% of DsRed+ neurons exhibited short-latency (≤0.2 ms) light-evoked potentials (*n* = 13/13 cells) (Figure 2C). This latency is too short to be mediated synaptically, and is indicative of ChR2 expression in the recorded neuron (Cruikshank et al., 2010). Thus, at least 61% of green fluorescent neurons express functional ChR2. Below we refer to green fluorescent cells as ChR2+ cells.

#### Abnormal morphology of ChR2 expressing axons

At postnatal day (P) 24, ChR2+ neurons and their axons had classical morphology, indistinguishable from rats in which only GFP was electroporated (Figure 1A). However, beginning at P33, neurons in ChR2-electroporated animals showed a progressive reduction in somatic fluorescence and the appearance of unusual fluorescent dots in regions containing ChR2-labeled axons. We term these “ChR2 puncta.” The number of ChR2 puncta increased progressively with age, and by P100 were very dense and present in all cortical layers (Figures 1B,C). In contrast, puncta were not apparent in animals electroporated with GFP alone (Figure 1D). Somatodendritic fluorescence remained limited to L2/3 neurons at all ages.

**Figure 3:**
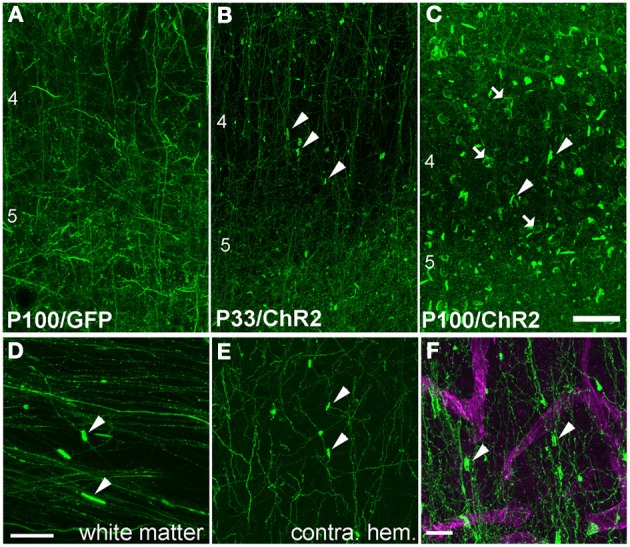
**Axonal swellings on ChR2+ axons. (A)** Morphologically normal L2/3 pyramidal cell axons in L4 and L5 of electroporated S1, from a P100 rat electroporated with GFP only. **(B–C)** ChR2+ axons in L4 and L5 of two ChR2-electroporated rats, at P33 and P100. Arrowheads, cylindrical axonal swellings. Arrows, calyceal structures. **(D–E)** Axonal swellings in white matter underlying electroporated S1, and in callosal S1 axons in contralateral (non-electroporated) S1. **(F)** Dual fluorescent staining of ChR+ axons in L4 and the capillary marker collagen IV (magenta). Scale bar in **(A–E)**, 50 μm. Scale bar in **(F)**, 10 μm.

Higher magnification imaging revealed that ChR2 puncta took two forms: elongated cylinders and large, round, calyx-like structures. Cylinders appeared earliest, were most numerous, and were found in layers 2–6, in the white matter underlying electroporated S1, and within the terminal field of callosally projecting expressing axons in contralateral S1. The lower density of fluorescent axons in the white matter and contralateral S1 allowed us to closely examine the cylinders, and to observe that each was connected to a fluorescently labeled axon (Figures 3D,E). In the white matter, the cylinders were oriented parallel to fasciculated axons (Figure 3D). Calyces developed later, were observed preferentially in layers 2–6, and were also connected to labeled axons (see below). These finding suggest that ChR2 puncta are axonal swellings or malformations.

**Figure 4:**
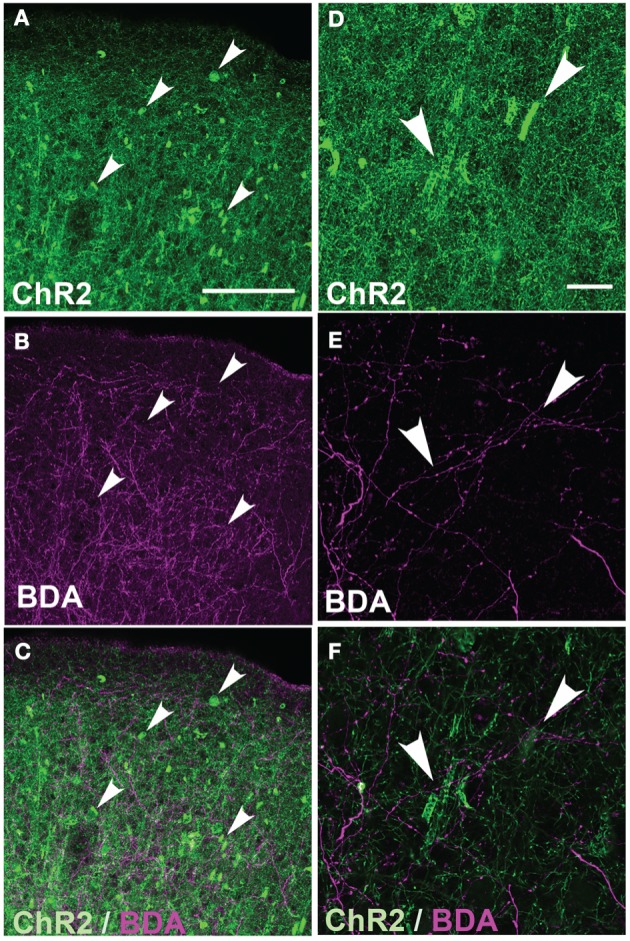
**Absence of axonal swellings on intermixed ChR2-negative axons. (A)** ChR2+ axons in S1 of P156 rat, showing numerous swellings. **(B)** Intermixed callosal axons from contralateral S1, anterogradely labeled by BDA injection and visualized with Alexa-647. Note absence of swellings. **(C)** Overlay. **(D–E)** Higher magnification of ChR2+ axons and BDA-labeled callosal axons. All images in **(A–E)** are maximum Z-projection confocal images. **(F)** Overlay of single confocal section of ChR2+ axons on top of image **(E)** of callosal axons. Scale bar is 500 μm for (in **A** for **A–C**) and 20 μm for (in **D** for **D–F**). Arrows show locations of ChR2-positive axonal swellings.

These axonal swellings could be due to expression of ChR2/GFP/DsRed or to damage due to electroporation itself. To distinguish these possibilities, we first tested whether puncta were visible in GFP-electroporated rats. No puncta were observed, up to P100 (Figure 3A). Second, we tested whether puncta in ChR2-electroporated animals were restricted to ChR2-expressing axons, or also appeared on intermixed ChR2-negative axons (Figure 4). In 3 rats, we electroporated ChR2/GFP/DsRed into left S1, and injected the anterograde tracer BDA into to right S1 of the same animals. After allowing 3–6 days for BDA transport, rats were perfused and BDA-labeled axons were visualized by staining with Alexa 647-conjugated streptavidin. ChR2+ axons in left S1 exhibited multiple axonal swellings, but intermixed BDA-labeled axons (shown in magenta in Figure 4) showed normal axonal structure. Thus, axonal swellings are specific to ChR2-expressing axons, and therefore are not a general effect of electroporation.

To test whether axonal abnormalities required light activation of ChR2, we compared the prevalence of ChR2-puncta in ChR2 animals that had received a fiber optic implant over S1 that delivered blue light (40 mW, 2 ms light pulse, 1000–1200 pulses per day, for ~30 days), versus animals with no blue light exposure. All animals were exposed to ambient room light on a 12 h light-dark cycle. The density of ChR2-puncta was indistinguishable in both cases (data not shown). Thus, ChR2-puncta formed either without ChR2 activation, or with minimal activation driven by spontaneous ChR2 gating or low-level room light exposure.

#### Fine structure of axonal cylinders and calyces

**Figure 5:**
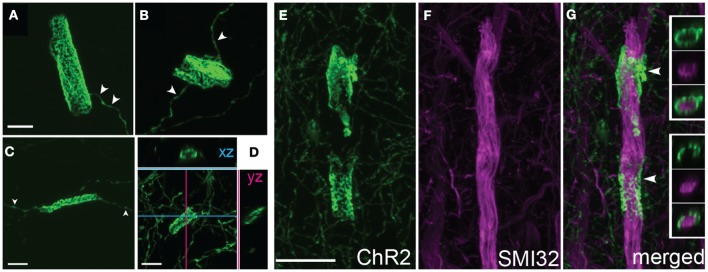
**Axonal cylinders. (A–C)** Example cylinders, showing patchy, mesh-like fluorescence. Arrowheads, single axon entering and exiting the cylinder. **(D)**, Confocal sectioning of a cylinder, demonstrating the hollow, tubular structure. Scale bar for **(A–D)**, 5 μm. **(E–G)**, Some axonal cylinders surround pyramidal cell apical dendrites stained by SMI32 antibody. Panels show ChR2+ cylinders in layer 4, co-staining with SMI32 antibody, and overlay showing ChR2+ membrane surrounding dendrite (arrowheads). Insets in **(G)**, single confocal optical sections showing ChR2 axon, SMI32, and overlay. Scale bar, 10 μm.

High magnification confocal microscopy revealed that cylinders were tubular structures with patchy, irregular ChR2-EYFP fluorescence. The patchy fluorescence may indicate a meshwork of axonal membrane, or aggregation of ChR2-EYFP protein within a continuous tube of membrane. Cylinders usually had a single axon entering and/or exiting (arrowheads in Figures 5A–C). Inner diameter was narrow (<5 μm), and length was variable ranging from 10 to 20 μm. Optical sectioning using confocal scanning revealed that tubes were hollow, and therefore may surround a central, unlabeled structure (Figures 5E, F).

**Figure 6:**
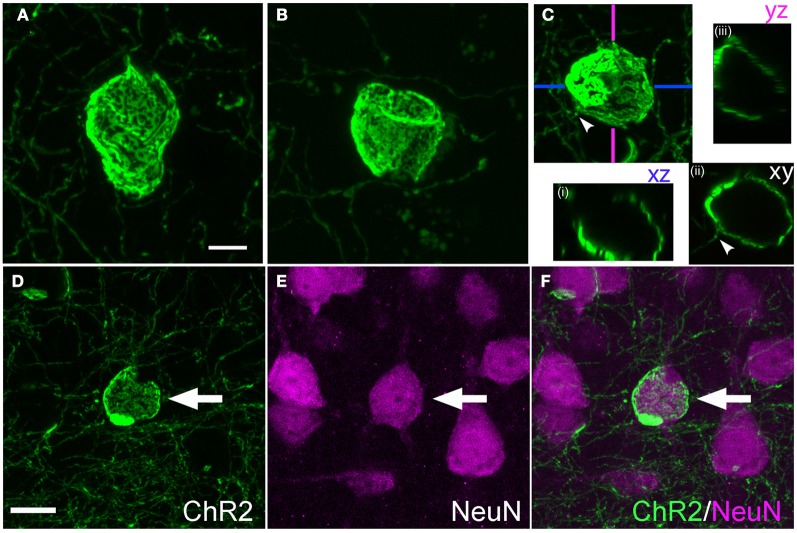
**Calyx-like structures. (A–C)**, Example calyces. Scale bar, 5 μm. Small panels in **(C)** are confocal optical sections demonstrating hollow structure with entering axon (arrowhead). **(D–F)**, Calyx structures surround neuronal somata. Panels show ChR2+ calyx, immunostaining for the neuronal marker Neu-N, and overlay. Scale bar, 10 μm.

Calyceal swellings had a diameter of 15–20 μm. Like cylinders, they typically contained bright and dark fluorescent patches, which may represent anastamosed axon segments or aggregations of ChR2-EYFP protein within a continuous membrane sheet (Figures 6A–C). A single axon often was observed entering or exiting the calyx (Figure 6C, arrowhead). Confocal x-y-z scans showed that calyces surrounded a central, round, unlabeled structure (Figure 6C).

#### Cylinders and calyces surround proximal apical dendrites and neuronal cell bodies

What target structures are enwrapped by axonal cylinders and calyces? In the case of cylinders, we first considered whether the target was capillaries, which can be ~5 μm diameter in cortex (Hirase et al., 2004; Michaloudi et al., 2006). Co-staining for the capillary marker Collagen type 4 confirmed that cylinders are not associated with capillaries (Figure 3F). We next tested whether cylinders colocalize with dendrites. We co-stained for SMI32, an anti-neurofilament antibody that stains apical dendrites of subpopulations of layer V pyramidal projection neurons. SMI32 labels L5 pyramidal neuron sparsely (~30%, Molnár and Cheung, 2006). Several cylinder structures were found to co-localize with, and enwrap, SMI32-positive apical dendrites (Figures 5E–G and **Movie 1**). For calyces, co-staining with neuronal cell body marker NeuN showed that calyx structures surround NeuN-positive somata (Figures 6D–F and **Movie 2**).

Thus, at least some tubes and calyces surround pyramidal cell apical dendritic trunks and neuronal cell bodies. This represents highly abnormal cellular targeting for L2/3 axons, which normally form sparse en passant synapses on basal, oblique, and distal apical dendrites of pyramidal cells, but not pyramidal cell somata.

#### Axonal morphology with viral expression of ChR2

**Figure 7:**
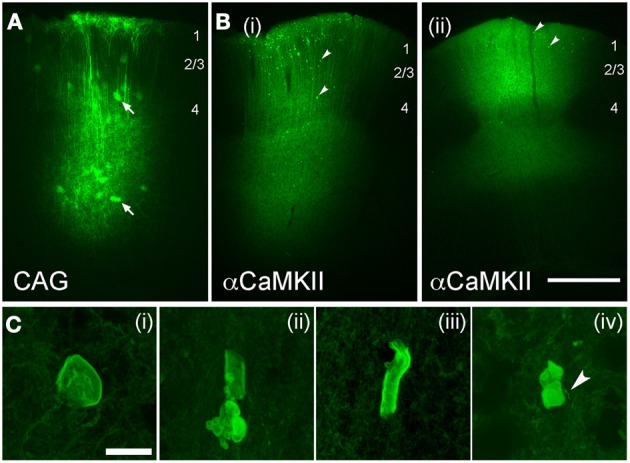
**Axonal swellings after viral-mediated expression of ChR2. (A,B)**, Low-power images of EYFP expression following long-term expression of AAV2.5-CAG-ChR2(H134R)-EYFP-WPRE (**A**; 80 days post-injection) and AAV2.5-αCaMKII-ChR2(H134R)-EYFP-WPRE [**B(i)** and **B(ii)**, 80 and 100 days post-injection, respectively]. Arrows in **(A)**, infected astrocytes. Arrowheads in **(B)**, ChR2 puncta. Scale bar for **(A,B)** is 500 μm. **(C)** Examples of axonal malformations in AAV2.5-αCaMKII cases. Arrowhead in **C(iv)**, entering/exiting axon. Scale bar, 10 μm.

Virus-mediated expression of ChR2 may be expected to drive fewer axonal abnormalities, since protein expression is typically lower than with IUE, and begins later in development. To test this, we expressed ChR2 (H134R) using either adeno-associated virus (AAV, serotype 2/5) or replication-incompetent lentivirus (Lenti) (Figure 7). Both ChR2 (H134R)-EYFP and ChR2 (H134R)-GFP were used. Virus was injected into L2/3-L4 of S1 at 30–40 days of age, and ChR2-EYFP labeled neurons were examined histologically 20–120 days later. Because fluorescence intensity was much lower than with IUE, visualization of ChR2-EYFP in individual axons required histological amplification using an anti-GFP antibody (which recognizes both ChR2-EYFP and ChR2-GFP proteins) and Alexa-488 secondary. We first tested AAV2.5 that expressed ChR2-GFP under the CAG promoter. ChR2-EYFP expression was observed in L2/3 and L5 pyramidal cells, astrocytes and some non-pyramidal neurons (presumed interneurons), as expected from the ubiquitous nature of the CAG promoter (Dittgen et al., 2004; Lawlor et al., 2009). Fluorescence intensity in ChR2-positive cells was substantially lower than for IUE cases, consistent with lower protein expression levels. No ChR2 puncta were observed either at 30, 45, 60, or 80 days post-injection (*n* = 2 cases per age) (Figure 7A).

To drive stronger expression, we used the excitatory neuron-specific αCaMKII promoter (Mayford et al., 1996) which has been reported to drive stronger expression than the CAG promoter for Lentivirus injections into S1 in mice at this age (Dittgen et al., 2004). AAV2.5/αCaMKII-ChR2-EYFP caused fluorescence expression in dendritic trunks and somata of L2/3 and L5 pyramidal cells at the injection site, and in axons projecting locally, in the subcortical white matter, contralateral S1, striatum and thalamus (consistent with known S1 projection targets, Aronoff et al., 2010). Fluorescence was localized to membranes, as expected for ChR2-EYFP signal. Dense neuropil staining prevented individual axons or small dendritic branches from being discerned at the injection site. ChR2 puncta were not observed at 20 and 30 days post-injection, but were clearly present at 80, 100, and 120 days post-injection (*n* = 2 animals per age). Two example cases are shown in Figure 7B.

ChR2 puncta in AAV/αCaMKII cases were different than for IUE. While some ChR2 puncta resembled axonal cylinders, most consisted of multi-lobed membrane “blebs,” many of which were associated with entering/exiting axons (arrow in Figure 7C). ChR2 puncta occurred in layers 2–6 at the injection site, but were absent in axonal projection fields in striatum, thalamus, and contralateral S1. Puncta were generally less prevalent than with IUE, and calyx-like structures were not observed. Sparse ChR2 puncta were also observed 60 days after injection of Lentivirus expressing ChR2 (H134R)-EYFP under the αCaMKII promoter. The viral expression results are summarized in Table 1. Thus, viral expression caused axonal malformations that increased with age (≥80 days) and predicted expression level (αCaMKII > CAG promoter), but were fewer than with IUE, and were limited to the injection site itself.

**Table 1:** **Axonal abnormalities after viral expression of ChR2-EYFP**.

**Viral vector**	**Promoter**	Survival (d)^*^	**Animals**	**ChR2 puncta?**
AAV2.5	CAG	30	2	None
		45	2	None
		60	2	None
		80	2	None
AAV2.5	αCaMKII	20	2	None
		30	2	None
		80	2	Dense (2/2 cases)
		100	2	Dense (1/2 cases)
		120	2	Dense (2/2 cases)
Lenti	αCaMKII	60	2	Sparse

* Days post-injection. All injections were at P30–40.

#### Abnormalities increase with expression level and age

**Figure 8:**
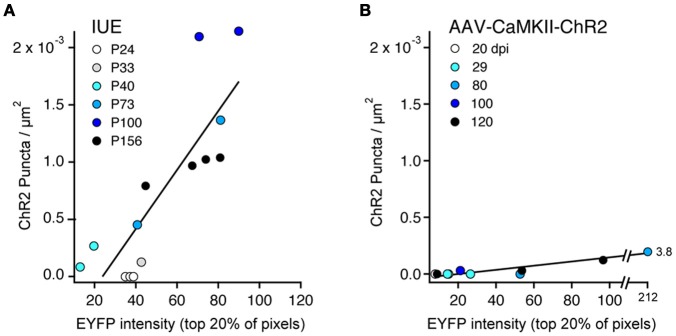
**ChR2 puncta density correlates with ChR2 expression level. (A)**, IUE cases. Each point represents one animal, analyzed in L2/3 at region of maximal S1 expression. Color indicates age. ChR2 expression is estimated from EYFP intensity, defined as the mean brightness value (range: 0–255) for the brightest 20% of pixels in the analysis region. Line is linear regression (*n* = 14 cases). With the exception of the youngest ages (P24–33), puncta density correlated strongly with ChR2 expression level. **(B)** AAV-CaMKII-ChR2 cases (*n* = 10 animals). Color indicates days post-injection (dpi).

We quantified the relationship between ChR2-EYFP expression level and ChR2 puncta in 14 IUE cases and 10 AAV-CaMKII-ChR2 cases. In each animal, we performed a confocal scan of L2/3 in the S1 region with the highest expression, using identical laser power and imaging parameters across animals. We used linear spectral unmixing to separate EYFP from GFP (present in IUE tissue) and Alexa-488 (present in AAV tissue after antibody-based amplification). Within a rectangular region of interest (~200 mm tangential width × ~500 mm radial height), we estimated ChR2 expression level as the mean EYFP fluorescence intensity of the brightest 20% of pixels. The 20% cutoff was chosen because only ~20% of L2/3 neurons express ChR2 (see above). The density of ChR2 puncta was measured in the same region. Within IUE cases, puncta density increased linearly with mean ChR2-EYFP expression level (*r* = 0.84) more strongly than with age (*r* = 0.60) (Figure 8A). Within AAV-CaMKII-ChR2 cases, puncta density also increased linearly with ChR2-EYFP expression (*r* = 0.94), but much less steeply than for IUE (Figure 8B). Thus, axonal abnormalities increased with expression level and age, and were more prevalent for IUE than AAV-mediated expression.

## Discussion

We observed structural abnormalities in L2/3 pyramidal cell axons after long-term expression of ChR2 *in vivo* using both *in utero* electroporation and viral expression. With highest ChR2 expression (achieved with IUE), these first appeared after 33 days, and consisted of axonal enlargements that formed hollow cylinders and calyx-like shells, which surrounded proximal apical dendrites and somata, respectively, of other neurons. Because L2/3 pyramidal cells do not normally form large contacts or synaptic endings onto the soma and proximal apical trunk (Feldmeyer et al., 2006; Broser et al., 2008; Bruno et al., 2009), these morphological changes may indicate altered subcellular targeting of axonal contacts. Their prevalence in L4 further suggests that L2/3 neurons may have formed an aberrant translaminar projection to L4, which is not normally a target of L2/3 neurons (Lübke and Feldmeyer, 2007; Lefort et al., 2009). Lower level expression using AAV and Lentiviral vectors reduced the incidence of axonal abnormalities, but abnormalities resembling membrane “blebs” were still observed with long-term, high-level expression (≥80 days) under the αCaMKII promoter. These findings raise concerns that long-term, high-level ChR2 expression has the potential, under some circumstances, to perturb structural features of cortical circuits.

### Axonal abnormalities reflect high-level, long-term expression

IUE drives strong, layer-specific expression of genes in cortical pyramidal cells (Saito and Nakatsuji, 2001; Tabata and Nakajima, 2001). Several prior studies used IUE to express GFP under the CAG promoter in L2/3 pyramidal cells, and found no abnormalities in axonal morphology at P4–6 (Cancedda et al., 2007), P15 (Mizuno et al., 2007, 2010; Sehara et al., 2010), P30 (Mizuno et al., 2010), or P60 (Wang et al., 2007). We also observed normal axonal morphology at P100 after GFP expression by IUE. Instead, abnormalities occurred only with ChR2 expression and survival periods >30 days (Figures 1, 3). Abnormalities did not occur on intermixed non-ChR2 expressing axons or with long-term expression of GFP rather than ChR2, and did not require light exposure greater than ambient room lighting, and are therefore likely to represent cell-autonomous effects of high-level ChR2 protein expression.

Axonal malformations increased with duration and level of ChR2 expression. For IUE-mediated expression, axonal abnormalities were absent at P24, sparse and small at P33, and increased dramatically in number and size by P100, correlated with ChR2 expression level (Figures 1, 3, 8). Viral vectors drove less ChR2-EYFP expression (judging by intensity of the EYFP signal), and substantially fewer axonal abnormalities. However, with sufficient duration of viral expression (>60–80 days), some ChR2 puncta were observed when using a strong promoter (αCaMKII) (Figures 7, 8). In contrast, the CAG promoter, which is reported to be a weaker viral promoter for cortical gene expression (Dittgen et al., 2004), did not drive detectable axonal malformations, even after 120 days of expression. We could not quantitatively compare ChR2 expression level under CaMKII vs. CAG promoters, because the available viruses contained different fluorescent proteins fused to ChR2. Axonal swellings were larger, more plentiful, and located in more distant axonal projection fields after IUE than after viral expression. The lower prevalence of abnormalities with viral expression may reflect either lower protein expression, later onset of expression, or both.

Previous studies used IUE of ChR2 under the CAG promoter to study the behavioral and circuit effects of activating L2/3 pyramidal cells in mice (Huber et al., 2008; Adesnik and Scanziani, 2010). In contrast to our results, normal neuronal morphology was reported after 8 months of expression (Huber et al., 2008). It is possible that rats (present study) are more susceptible to ChR2-induced axonal abnormalities than mice (prior studies). Alternatively, subtle changes in axonal morphology may have been missed.

### Malformations reveal cellular and laminar mistargeting of L2/3 axons

The robust abnormalities we observed following IUE demonstrate subcellular and laminar mistargeting of L2/3 axons. ChR2+ calyces were physically associated with NeuN-labeled neuronal somata (e.g., Figure 6), and were not found in the white matter, suggesting that they represent grossly enlarged contact zones onto target neuronal cell bodies. The target(s) of axonal cylinders are less clear. A small fraction of cylinders enclosed SMI32-positive dendrites, which marks proximal apical dendrites in a subset of L5 thick-tufted pyramidal cells (Figure 5). The targets of cylinders that did not co-localize with SMI32 are unknown, but we speculate that these target other neuronal dendrites, and that the small number of cylinders in white matter may target dendrites of white matter GABAergic neurons (Tomioka and Rockland, 2007).

We do not know if axonal malformations in IUE cases represent synapses. L2/3 pyramidal cells normally make isolated, en passant boutons onto secondary or higher-order dendrites, not large synaptic endings onto the soma or proximal apical trunk (Feldmeyer et al., 2006; Broser et al., 2008; Bruno et al., 2009). Thus, axonal calyces and cylinders represent abnormal subcellular targeting of axonal contacts, as well as abnormal morphology. Moreover, because many calyces were found in L4, which is not normally a target of L2/3 pyramidal cells, ChR2 expression may drive formation of an abnormal translaminar projection (L2/3 → L4). Thus, these findings raise concerns that long-term expression of ChR2 may alter cortical circuit connectivity. Axonal swellings in viral expression cases were typically membrane blebs rather than hollow cylinders or calyces, and were confined to the injection site center, suggesting relatively minor structural alterations, not functional circuit changes. However, it is important to emphasize that we do not know whether the structural abnormalities observed here, even for IUE, are associated with any functional changes in circuit physiology.

### Molecular mechanisms of axonal malformation are unknown

The mechanisms by which ChR2-EYFP overexpression drive axonal malformations are not known. In principal, malformations may be caused by overexpression of any axon-localized membrane protein, via distortion of local membrane shape or interference with endogenous protein interactions. Alternatively, malformations may by driven by abnormal calcium signaling, due to calcium permeation through the ChR2 channel (Nagel et al., 2003). Though light-evoked channel gating was not necessary for malformations, spontaneous gating (or gating by ambient room light) could generate sufficient axonal calcium to interfere with calcium-dependent axon guidance (Tojima et al., 2011), synapse targeting, or morphological synapse plasticity (Patterson and Yasuda, 2011). Supporting this possibility, a mis-sense mutation that increases calcium flux through TRPV4 channels is associated with several axonal neuropathies (Jang et al., 2012).

## Summary

Long-term neuronal expression of ChR2 and other optogenetic constructs is an essential method to study the circuit and cellular bases for behavior. It is also a promising therapeutic strategy for some neurological disorders in humans (Zhang et al., 2007; Bernstein and Boyden, 2011; Fenno et al., 2011). Our results show that long-term, high-level expression of ChR2 in rat neocortex can, under some conditions, substantially perturb axonal structure, axonal targeting, and layer-to-layer wiring specificity of cortical circuits. These changes were not observed in other long-term studies (Huber et al., 2008). Viral expression produced many fewer abnormalities than IUE. These findings indicate that (1) the lowest possible expression levels should be used for long-term ChR2 expression; and (2) careful histological examination should be used to verify normal morphology and connectivity of ChR2-expressing neural circuits, particularly for studies involving long-term or high-level expression.

### Conflict of interest statement

The authors declare that the research was conducted in the absence of any commercial or financial relationships that could be construed as a potential conflict of interest.

## References

[B1] Adesnik H. Scanziani M. (2010). Lateral competition for cortical space by layer-specific horizontal circuits. Nature 464, 1155–1160 10.1038/nature08935 20414303 PMC2908490

[B1a] Allen C. B. Celikel T. Feldman D. E. (2003). Long-term depression induced by sensory deprivation during cortical map plasticity *in vivo*. Nat. Neurosci. 6, 291–299 10.1038/nn1012 12577061

[B2] Aronoff R. Matyas F. Mateo C. Ciron C. Schneider B. Petersen C. C. (2010). Long-range connectivity of mouse primary somatosensory barrel cortex. Eur. J. Neurosci. 31, 2221–2233 10.1111/j.1460-9568.2010.07264.x 20550566

[B3] Bernstein J. G. Boyden E. S. (2011). Optogenetic tools for analyzing the neural circuits of behavior. Trends Cogn. Sci. 15, 592–600 10.1016/j.tics.2011.10.003 22055387 PMC3225502

[B4] Broser P. Grinevich V. Osten P. Sakmann B. Wallace D. J. (2008). Critical period plasticity of axonal arbors of layer 2/3 pyramidal neurons in rat somatosensory cortex: layer-specific reduction of projections into deprived cortical columns. Cereb. Cortex 18, 1588–1603 10.1093/cercor/bhm189 17998276 PMC2430153

[B5] Bruno R. M. Hahn T. T. Wallace D. J. de Kock C. P. Sakmann B. (2009). Sensory experience alters specific branches of individual corticocortical axons during development. J. Neurosci. 29, 3172–3181 10.1523/JNEUROSCI.5911-08.2009 19279254 PMC6666463

[B6] Cancedda L. Fiumelli H. Chen K. Poo M. M. (2007). Excitatory GABA action is essential for morphological maturation of cortical neurons *in vivo*. J. Neurosci. 27, 5224–5235 10.1523/JNEUROSCI.5169-06.2007 17494709 PMC6672363

[B6a] Cruikshank S. J. Urabe H. Nurmikko A. V. Connors B. W. (2010). Pathway-specific feedforward circuits between thalamus and neocortex revealed by selective optical stimulation of axons. Neuron 65, 230–245 10.1016/j.neuron.2009.12.025 20152129 PMC2826223

[B7] Dittgen T. Nimmerjahn A. Komai S. Licznerski P. Waters J. Margrie T. W. (2004). Lentivirus-based genetic manipulations of cortical neurons and their optical and electrophysiological monitoring *in vivo*. Proc. Natl. Acad. Sci. U.S.A. 101, 18206–18211 10.1073/pnas.0407976101 15608064 PMC539748

[B8] Feldmeyer D. Lübke J. Sakmann B. (2006). Efficacy and connectivity of intracolumnar pairs of layer 2/3 pyramidal cells in the barrel cortex of juvenile rats. J. Physiol. 575, 583–602 10.1113/jphysiol.2006.105106 16793907 PMC1819447

[B9] Fenno L. Yizhar O. Deisseroth K. (2011). The development and application of optogenetics. Annu. Rev. Neurosci. 34, 389–412 10.1146/annurev-neuro-061010-113817 21692661 PMC6699620

[B10] Hatanaka Y. Hisanaga S. Heizmann C. W. Murakami F. (2004). Distinct migratory behavior of early- and late-born neurons derived from the cortical ventricular zone. J. Comp. Neurol. 479, 1–14 10.1002/cne.20256 15389616

[B11] Hirase H. Creso J. Buzsáki G. (2004). Capillary level imaging of local cerebral blood flow in bicuculline-induced epileptic foci. Neuroscience 128, 209–216 10.1016/j.neuroscience.2004.07.002 15450368

[B12] Huber D. Petreanu L. Ghitani N. Ranade S. Hromádka T. Mainen Z. (2008). Sparse optical microstimulation in barrel cortex drives learned behaviour in freely moving mice. Nature 451, 61–64 10.1038/nature06445 18094685 PMC3425380

[B13] Jang Y. Jung J. Kim H. Oh J. Jeon J. H. Jung S. (2012). Axonal neuropathy-associated TRPV4 regulates neurotrophic factor-derived axonal growth. J. Biol. Chem. 287, 6014–6024 10.1074/jbc.M111.316315 22187434 PMC3285368

[B14] Lawlor P. A. Bland R. J. Mouravlev A. Young D. During M. J. (2009). Efficient gene delivery and selective transduction of glial cells in the mammalian brain by AAV serotypes isolated from nonhuman primates. Mol. Ther. 17, 1692–1702 10.1038/mt.2009.170 19638961 PMC2835020

[B15] Lefort S. Tomm C. Floyd Sarria J. C. Petersen C. C. (2009). The excitatory neuronal network of the C2 barrel column in mouse primary somatosensory cortex. Neuron 61, 301–316 10.1016/j.neuron.2008.12.020 19186171

[B16] Lübke J. Feldmeyer D. (2007). Excitatory signal flow and connectivity in a cortical column: focus on barrel cortex. Brain Struct. Funct. 212, 3–17 10.1007/s00429-007-0144-2 17717695

[B17] Mayford M. Bach M. E. Huang Y. Y. Wang L. Hawkins R. D. Kandel E. R. (1996). Control of memory formation through regulated expression of a CaMKII transgene. Science 274, 1678–1683 10.1126/science.274.5293.1678 8939850

[B18] Michaloudi H. Batzios C. Grivas I. Chiotelli M. Papadopoulos G. C. (2006). Developmental changes in the vascular network of the rat visual areas 17, 18 and 18a. Brain Res. 1103, 1–12 10.1016/j.brainres.2006.05.069 16806119

[B19] Miyashita T. Wintzer M. Kurotani T. Konishi T. Ichinohe N. Rockland K. S. (2010). Neurotrophin-3 is involved in the formation of apical dendritic bundles in cortical layer 2 of the rat. Cereb. Cortex 20, 229–240 10.1093/cercor/bhp093 19447860 PMC2792193

[B20] Mizuno H. Hirano T. Tagawa Y. (2007). Evidence for activity-dependent cortical wiring: formation of interhemispheric connections in neonatal mouse visual cortex requires projection neuron activity. J. Neurosci. 27, 6760–6770 10.1523/JNEUROSCI.1215-07.2007 17581963 PMC6672694

[B21] Mizuno H. Hirano T. Tagawa Y. (2010). Pre-synaptic and post-synaptic neuronal activity supports the axon development of callosal projection neurons during different post-natal periods in the mouse cerebral cortex. Eur. J. Neurosci. 31, 410–424 10.1111/j.1460-9568.2009.07070.x 20105242

[B22] Molnár Z. Cheung A. F. (2006). Towards the classification of subpopulations of layer V pyramidal projection neurons. Neurosci. Res. 55, 105–115 10.1016/j.neures.2006.02.008 16542744

[B23] Nagel G. Szellas T. Huhn W. Kateriya S. Adeishvili N. Berthold P. (2003). Channelrhodopsin-2, a directly light-gated cation-selective membrane channel. Proc. Natl. Acad. Sci. U.S.A. 100, 13940–13945 10.1073/pnas.1936192100 14615590 PMC283525

[B24] Niwa H. Yamamura K. Miyazaki J. (1991). Efficient selection for high-expression transfectants with a novel eukaryotic vector. Gene 108, 193–199 1660837 10.1016/0378-1119(91)90434-d

[B25] Patterson M. Yasuda R. (2011). Signalling pathways underlying structural plasticity of dendritic spines. Br. J. Pharmacol. 163, 1626–1638 10.1111/j.1476-5381.2011.01328.x 21410464 PMC3166652

[B26] Petreanu L. Mao T. Sternson S. M. Svoboda K. (2009). The subcellular organization of neocortical excitatory connections. Nature 457, 1142–1145 10.1038/nature07709 19151697 PMC2745650

[B27] Saito T. Nakatsuji N. (2001). Efficient gene transfer into the embryonic mouse brain using *in vivo* electroporation. Dev. Biol. 240, 237–246 10.1006/dbio.2001.0439 11784059

[B28] Sehara K. Toda T. Iwai L. Wakimoto M. Tanno K. Matsubayashi Y. (2010). Whisker-related axonal patterns and plasticity of layer 2/3 neurons in the mouse barrel cortex. J. Neurosci. 30, 3082–3092 10.1523/JNEUROSCI.6096-09.2010 20181605 PMC6633930

[B29] Tabata H. Nakajima K. (2001). Efficient *in utero* gene transfer system to the developing mouse brain using electroporation: visualization of neuronal migration in the developing cortex. Neuroscience 103, 865–872 10.1016/S0306-4522(01)00016-1 11301197

[B30] Tojima T. Hines J. H. Henley J. R. Kamiguchi H. (2011). Second messengers and membrane trafficking direct and organize growth cone steering. Nat. Rev. Neurosci. 12, 191–203 10.1038/nrn2996 21386859 PMC3133775

[B31] Tomioka R. Rockland K. S. (2007). Long-distance corticocortical GABAergic neurons in the adult monkey white and gray matter. J. Comp. Neurol. 505, 526–538 10.1002/cne.21504 17924571

[B32] Toni N. Laplagne D. A. Zhao C. Lombardi G. Ribak C. E. Gage F. H. (2008). Neurons born in the adult dentate gyrus form functional synapses with target cells. Nat. Neurosci. 11, 901–907 10.1038/nn.2156 18622400 PMC2572641

[B33] Wang C. L. Zhang L. Zhou Y. Zhou J. Yang X. J. Duan S. M. (2007). Activity-dependent development of callosal projections in the somatosensory cortex. J. Neurosci. 27, 11334–11342 10.1523/JNEUROSCI.3380-07.2007 17942728 PMC6673014

[B34] Zhang F. Aravanis A. M. Adamantidis A. de Lecea L. Deisseroth K. (2007). Circuit-breakers: optical technologies for probing neural signals and systems. Nat. Rev. Neurosci. 8, 577–581 10.1038/nrn2192 17643087

