# Corrigendum: Long-term channelrhodopsin-2 (ChR2) expression can induce abnormal axonal morphology and targeting in cerebral cortex

**DOI:** 10.3389/fncir.2013.00045

**Published:** 2013-03-20

**Authors:** Toshio Miyashita, Yu R. Shao, Jason Chung, Olivia Pourzia, Daniel E. Feldman

**Affiliations:** Department of Molecular and Cell Biology, University of CaliforniaBerkeley, CA, USA

In our recent paper (Miyashita et al., 2013), we showed that long-term, high-level channelrhodopsin-2 (ChR2) expression by *in utero* electroporation (IUE) produces structural abnormalities in the axons of ChR2-expressing pyramidal cells in rat somatosensory cortex. In the Discussion of our paper, we mentioned that such abnormalities were not observed in an earlier study using long-term IUE of ChR2 under the same promoter (Huber et al., 2008). A methodological difference has been brought to our attention that likely explains this difference. Huber et al. expressed wildtype ChR2 (Chop2-315 from Nagel et al., 2003), while we expressed hChR2 that was codon-optimized for higher mammalian expression (Zhang et al., 2006). This suggests that lower ChR2 protein levels in the Huber study may have enabled long-term expression without axonal malformations. This further supports our main conclusion that morphological abnormalities are associated with high-level, long-term expression of ChR2 protein, and that lower level expression is necessary for long-term studies.
